# New one-pot synthesis of Au and Ag nanoparticles using green rust reactive particle as a micro-reactor

**DOI:** 10.1186/1556-276X-8-95

**Published:** 2013-02-22

**Authors:** Sondra Ayadi, Cristian Perca, Ludovic Legrand

**Affiliations:** 1 LAMBE UMR8587, CNRS-CEA-Université Evry, 1, rue du Père Jarlan, Evry Cedex 91025, France

**Keywords:** Gold, Green rust, Micro-reactor, Nanoparticles, Silver

## Abstract

A new, simple, and fast one-pot synthesis of supported Au or Ag nanoparticles is implemented, for which a reactive Fe(II)-bearing green rust inorganic particle is used as an individual micro-reactor acting as both the reducing agent and support for the resulting metal nanoparticles. The mechanism involves both the solid-state oxidation of the green rust support (sulfate or carbonate) and the reduction-precipitation of soluble metal precursor. The resulting nanohybrids have a platy inorganic part supporting about one to ten nanoparticles with sizes in the 20 to 120 nm range.

## Background

Nanoparticles of noble metals exhibit unique optical, chemical, catalytic, and electronic properties which make them attractive for a wide range of applications in many domains. The most common way for preparing such nanoparticles, named as ‘wet chemistry’, consists in reducing a soluble metal precursor (Au^III^ or Ag^I^) by a soluble reducing agent in the presence of a stabilizing species which keeps the formed nanoparticles from aggregation. Turkevich-Fens’s method uses AuCl_4_^−^ ions and sodium citrate as both reducer and stabilizing agent and gives approximately 20-nm spherical nanoparticles [1,2]. Numerous other stabilizing agents have been further used. In Brust’s synthesis, a two-phase aqueous-organic solution with tetraoctylammonium bromide transfer species and a strong stabilizing thiol agent are implemented and the reaction of AuCl_4_^−^ and NaBH_4_ in these conditions allows the preparation of stable 1- to 5-nm Au clusters [3]. Regarding silver nanoparticles, the most common synthesis is the reduction of silver cation/complex by chemical agents such as borohydride or hydrazine [4,5]. From the so-called polyol process displaying ethylene glycol as both reductant and solvent, various nanoparticles including Au and Ag could be obtained [6,7]. As hazardous products occur and may generate biocompatibility or environment problems, a recent development of ‘green synthesis’ was stimulated, for which environmentally friendly reducing agents are used, including saccharides or natural extracts [8]. Suspensions of supported metal nanoparticles on inorganic solids can be formed by wetness impregnation or alkaline (co-) precipitation [9,10]. These routes give low metal loads (wt.%) and require a final gas reduction treatment by H_2_ or CO, with some possible efficiency problems for the complete conversion to metal.

Fe^2+^ ion is a ‘green’ reducing species present in the crystalline structure of various solids including sulfides, carbonates, hydroxisalts, and clays. As the oxidation of structural Fe^II^ ions usually occurs in a very cathodic potential domain, the transfer of electrons to numerous oxidants is therefore possible. The reduction of Ag^I^ and Au^III^ soluble species by iron sulfide minerals has already been reported [11,12]. However, the mineral samples available for laboratory experiments usually display very large dimensions, which preclude any potential applications.

Green rusts (GR) are layered Fe^II^-Fe^III^ hydroxisalts composed of positively charged Fe(OH)_6_ octahedra sheets alternating with interlayers filled with charge-compensating anions and water molecules [13]. Early studies on the reduction of Ag^I^ or Au^III^ by green rusts were reported in 2003, from Heasmann et al. and O’Loughlin et al. [14,15]. The presence of Au or Ag metal was evidenced by X-ray absorption spectroscopy and transmission electron microscopy. Later, these green rusts doped with very low metal loads were utilized as reducing compounds for the removal of some chlorinated hydrocarbons [16,17]. In these studies, the reaction mechanisms between green rust and soluble metal precursor were not detailed and none of the studies gave an evidence of metallic particles by X-ray diffraction (XRD). The proposed mechanism involves the oxidation of sulfate green rust into magnetite Fe_3_O_4_, coupled to the reduction of Au^III^ or Ag^I^ to Au or Ag.

The oxidation mechanisms of green rusts have been extensively studied. This reaction can imply transformations via solution, i.e., dissolution, oxidation, and precipitation of the resulting ferric oxy-hydroxides, lepidocrocite, and goethite [18,19]. Otherwise, a solid-state oxidation of green rusts involving both the conversion of Fe^II^ to Fe^III^ inside the crystal lattice and the charge-compensating loss of protons is also possible [19-22]. The latter mechanism especially occurs when high oxidation rate is imposed, for example, by reaction with some soluble oxidizers such as H_2_O_2_. The resulting ferric products, named as ‘exGR-Fe(III)’ or as ‘ferric green rust’, keep the same apparent morphology as the initial green rusts; only local disorders at nanometric scale are induced, as indicated by the disappearance or the large decrease of (00l) lines in diffraction patterns [19,21,22].

In the present paper, we introduce a new one-pot synthesis of supported noble metal nanoparticles in which the green rust particle is an individual micro-reactor acting as both the reducing agent and the support for the resulting metal nanoparticles. Carbonate (GRc) or sulfate (GRs) green rust suspensions were obtained from the oxidation by air of slightly alkaline solutions containing ferrous species and carbonate or sulfate anions and the reactions with Au^III^ or Ag^I^ were operated shortly after, in the same solution [23]. Our purpose is the production of Au or Ag nanoparticles by this new method and we therefore target high metal loads. This simple synthesis is carried out at near ambient temperature, in aqueous solution, and requires only common salts; it is environment friendly since no organic solvents/additives are used and the filtrates do not represent a problem for recycling.

## Methods

### Chemicals

Sodium bicarbonate NaHCO_3_, sodium sulfate Na_2_SO_4_,10H_2_O, iron chloride FeCl_2_,4H_2_0 (>99%), iron sulfate FeSO_4_,10H_2_O (>99%), potassium tetrachloroaurate KAuCl_4_ (98%), silver nitrate AgNO_3_ (>99%), ammoniac NH_3_(aq) (33%), sodium hydroxide NaOH (10 M solution) purchased from Sigma Aldrich (Steinheim, Germany). The solutions were prepared with 18 MΩ cm nanopure water.

#### Solution reactions

For the synthesis of carbonate (or sulfate) green rusts, 50 ml of 0.4 M NaHCO_3_ (or 0.4 M Na_2_SO_4_) solution is put into a cylindrical glass cell thermostated at 25°C and stirred at 300 rpm under argon for 15 min. Then, 0.5 ml of 1 M FeCl_2_ solution or 1 M FeSO_4_ solution is introduced and 10 M NaOH solution is added dropwise to fix the initial pH at a value of 9.5. Finally, argon bubbling is stopped and the cell is opened to air. After about 25 min, a green rust suspension containing 333 μmol Fe^II^ is obtained. The Au^III^ solution contains 0.05 M KAuCl_4_; the Ag^I^ solution contains 0.1 M Ag(NH_3_)_2_^+^ and 0.3 M NH_3_. The reactions with green rust suspensions are conducted by adding an appropriate quantity of Au^III^ or Ag^I^, expressed as a stoichiometric ratio *R*; *R* = 100% corresponds to 111 μmol Au^III^ or to 333 μmol Ag^I^. The solution reactions are monitored by recording redox potential with a WTW multimeter, using a platinum working electrode (Radiometer Tacussel, La Fontaine du Vaisseau, Neuilly Plaisance, France) and a homemade AgCl/Ag-0.1 M NaCl reference electrode (0.23 V with respect to standard hydrogen electrode).

### Characterization

The resulting metal-inorganic nanohybrids were characterized by Fourier transform infrared spectroscopy (FTIR) spectrometry, X-ray diffraction, and scanning electron microscopy. After interactions of about 20 to 30 min, solid samples were separated by filtration, carefully rinsed with deionised water, and dried at ambient temperature for at least 24 h. They were weighted and then characterized. FTIR data were recorded on a Bruker IFS 28 spectrometer (Bruker optics, Wissembourg, France). Powder samples were pressed to pellets with KBr and analyzed by direct transmission mode. XRD measurements were carried out using a Bruker D8 diffractometer with CuK_α_ radiation (1.5406 Å). Scanning electron microscope (SEM) examinations were performed by a LEO 1530 (Carl Zeiss AG, Oberkochen, Germany) microscope using in-lens and backscattered electron modes.

## Results and discussion

**Figure 1:**
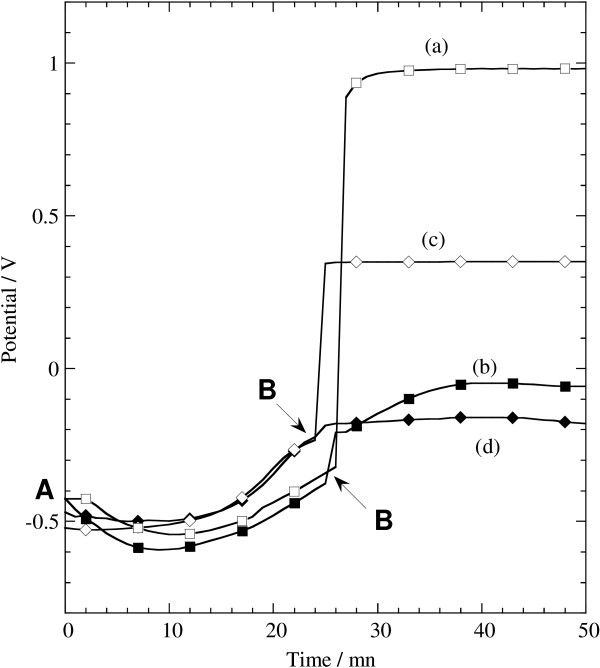
**Potential-time transients. **Synthesis of green rust suspension from point A to point B and its further reaction with the soluble metal precursor which is added at point B at various stoichiometric ratios *R*; sulfate green rust and Au^III^, **(a) ***R* = 120% and **(b) ***R* = 25%; carbonate green rust and Ag^I ^**(c) ***R* = 120% and **(d) ***R* = 15.

Figure 1 displays potential-time transients recorded during the synthesis of green rust suspension (from point A to point B) and its reaction, beyond point B, with the soluble metal precursor, Au^III^ (curves a and b) or Ag^I^ (curves c and d). The formation of pure carbonate or sulfate green rust suspensions at points B was confirmed by FTIR analysis. Total Fe^II^ titrations done at points B gave values near 67% of the initial Fe^II^ quantity, consistently with the formula of carbonate or sulfate green rusts, Fe^II^_4_Fe^III^_2_(OH)_12_CO_3_,2H_2_O or Fe^II^_4_Fe^III^_2_(OH)_12_SO_4_,8H_2_O [19,24]. We also checked that the quantity of Fe^II^ remaining in the solution at point B was negligible. At point B, the cell was closed and put under argon bubbling. As soon as the soluble metal precursor was introduced, a sharp increase of potential is observed, suggesting that the reaction quickly reaches completion. When an excess of soluble metal precursor with respect to Fe^II^ is added (stoichiometry ratio *R* > 100%), the potential stabilizes at a value that is consistent with Au^III^/Au or Ag^I^/Ag redox systems, AuCl_4_^−^/Au (E° = 1.00 V/ESH) for curve a and Ag(NH_3_)_2_^+^/Ag (E° = 0.37 V/ESH) for curve c. Otherwise (*R* < 100%), the lower potential values beyond point B in curves b and d are related to Fe^II^ and Fe^III^ species. In this case, after removing the solid sample from the solution, the contact with air provokes the oxidation of the remaining green rust.

**Figure 2:**
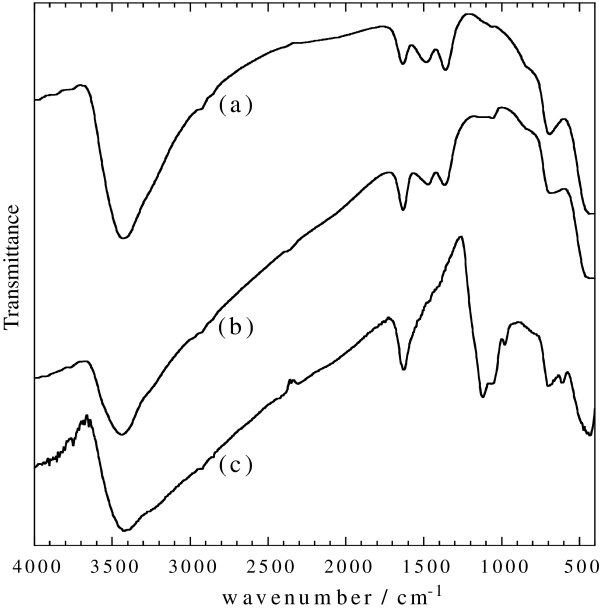
**FTIR spectra of the solid samples. **Solid samples obtained after reaction between **(a) **GRc and Ag^I^, *R* = 100%, **(b) **GRc and Au^III^, *R* = 200%, and **(c) **GRs and Au^III^, *R* = 150%. The ferric product exGRc-Fe(III) resulting from the solid-state oxidation of carbonate green rust exhibits bands at 450, 695, and 850 (sh), 1,065, 1,485, and 1,530 (sh), and 1,640, 3,200 and 3,430 cm^−1^.The ferric product exGRs-Fe(III) resulting from the solid-state oxidation of sulfate green rust exhibits bands at 450, 605, 700, 980, 1,055, 1,120, and 1,200 (sh), and 1,640, 3,220 and 3,420 cm^−1^.

The FTIR spectra of the solid samples obtained after the reaction of carbonate green rust with Ag^I^ or Au^III^ are similar and exhibit bands corresponding to exGRc-Fe(III), the ferric product resulting from the solid-state oxidation of carbonate green rust (spectra a and b in Figure 2) [22]. A similar solid-state oxidation leading to exGRs-Fe(III) also occurs when using sulfate green rust. No other characteristic bands are obviously observed, suggesting the absence of any other iron compounds.

**Figure 3:**
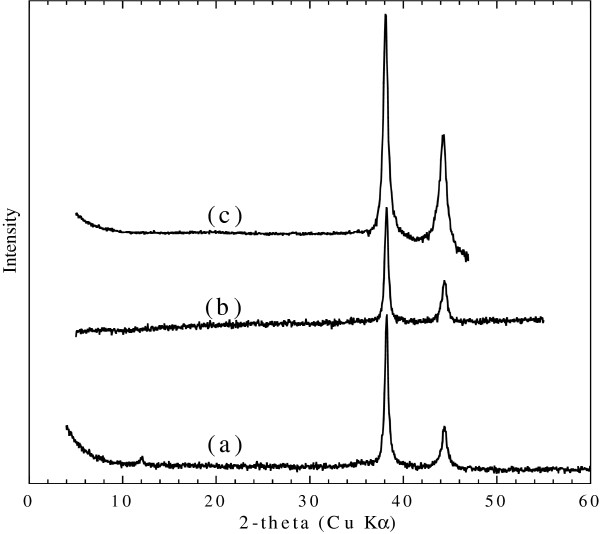
**X-ray diffractograms of the solid samples. **Solid samples obtained after reaction between **(a) **GRc and Ag^I^, *R* = 100% **(b) **GRc and Au^III^, *R* = 200% and **(c) **GRs and Au^III^, *R* = 120%. JCPDS cards are 00-004-0783 for silver Ag and 00-004-0784 for gold Au.

Figure 3 gives the XRD patterns of the solid samples resulting from the interaction between Au^III^/GRc (curve a), Au^III^/GRs (curve b), and Ag^I^/GRs (curve c). In the XRD patterns of the solid samples, the formation of Au metal or Ag metal is evidenced by their (111) and (200) lines with 2*θ* values at 38.2° and 44.4 or 38.1° and 44.2°. The size *s* of X-ray coherent domains was determined from the two diffraction lines according to the simplified Scherrer equation (Equation 1) with the value of 20 to 14 nm for Au^III^/GRc, 18 to 12 nm for Au^III^/GRs, and 14 to 10 nm for Ag^I^/GRs:

(1) $$s=\frac{0.89\lambda }{B\cos\;\theta },$$

where *s* is the size of X-ray coherent domains (nm); *B*, the angular width at half-height (rad); *θ*, the Bragg’s law diffraction angle; and *λ*, the X-ray wavelength (nm).

In pattern a, the low intensity line at 2*θ* = 12.05° confirms the presence of exGRc-Fe(III) ferric product [19,23]. A similar line is not observed for exGRs-Fe(III), because the particles are more susceptible to oxidation-induced disorder due to lower thickness and larger initial interplanar distance [22]. Note that magnetite, as an oxidation product, is not detected, contrary to what was reported by O’Loughlin or Choi [15,17]. Considering the following formula for carbonate green rust, GRc = Fe^II^_4_Fe^III^_2_(OH)_12_CO_3_,2H_2_O and sulfate green rust, GRs = Fe^II^_4_Fe^III^_2_(OH)_12_SO_4_,8H_2_O, the following schematic reactions can be proposed:

(2) $$\begin{array}{l} \frac{1}{4}\left\{\text{GRc}\;\text{or}\;\text{GRs}\right\}\;+\;\text{A}g^{I}\;\to \;\frac{1}{4}\left\{\text{exGRc}-\text{Fe}\left(\text{III}\right)\right)\;\text{or}\\ \left(\;\text{exGRs}-\text{Fe}\left(\text{III}\right)\right\}\;+\;\text{Ag} \end{array}$$

(3) $$\begin{array}{l} \frac{3}{4}\left\{\text{GRc}\;\text{or}\;\text{GRs}\right\}\;+\;\text{A}{\text{u}}^{\text{III}}\;\to \;\frac{3}{4}\left\{\text{exGRc}-\text{Fe}\left(\text{III}\right)\;\text{or}\right)\\ \left(\;\text{exGRs}-\text{Fe}\left(\text{III}\right)\right\}\;+\;\text{Au} \end{array}$$

**Figure 4:**
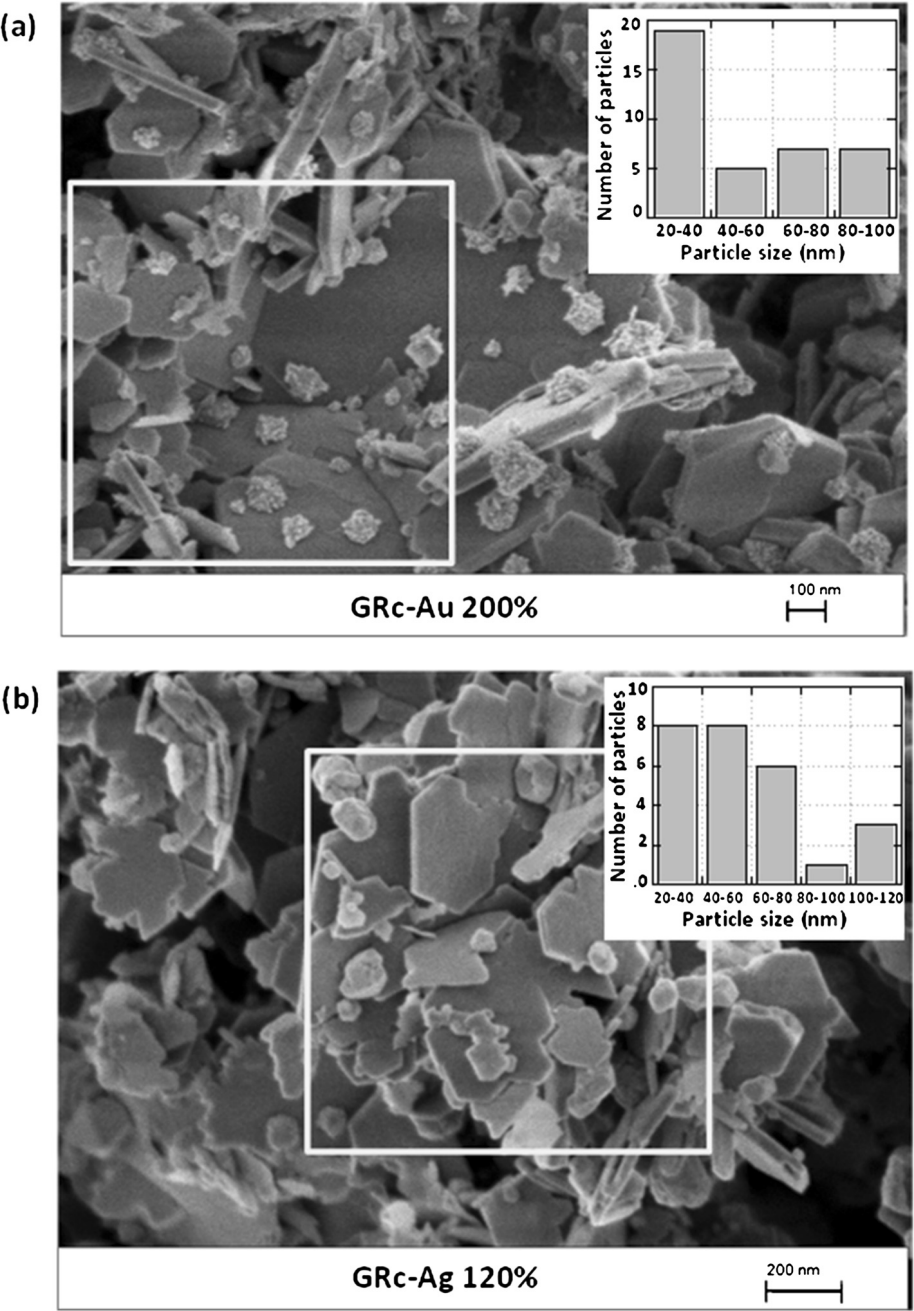
**In-lens SEM microscopy pictures. **Solid samples obtained after reaction between (**a**) GRc and Au^III^, *R* = 200% and (**b**) GRc and Ag^I^, *R* = 120%. Size distribution histograms are given in the inserts (a) 1 μm^2^, 38 Au nanoparticles and (b) 1 μm^2^, 26 Ag nanoparticles.

In order to determine the morphology of the samples resulting from the interaction of green rust and metal precursors, in-lens mode SEM analysis was performed. On both pictures of Figure 4, exGRc-Fe(III) appears as platy particles of several hundred nanometers in diameter and several tenth nanometers in thickness, mostly hexagonal in shape; this result was fully expected since the solid-state oxidation of carbonate green rust does not change the morphology of the particles [19]. In Figure 4a, Au nanoparticles are present as flattened hemispherical clusters comprising several individual nanocrystallites. The size of these little nanocrystallites, about 10 to 15 nm, is consistent with the *d* values of X-ray coherent domains given above. Au nanoparticles are preferentially deposited onto the flat faces of inorganic particles, rather than onto their sides. The insert reports the distribution of metal nanoparticles worked out from the count and the determination of diameter values performed within the 1 μm^2^ surface area open square. The obtained surface density of particles, *N*_Au_, is 38 μm^−2^. Assuming that Au nanoparticles are hemispheres, the total volume of Au was assessed from the distribution given in the insert and after applying a two thirds correction factor in order to take into account the flattened shape of nanoparticles, *V*_Au_ = 1.5 × 10^−15^ cm^3^. Then according to Equation 3 and assuming that the molar mass and density of exGRc-Fe(III) are very close to the ones of GRc, at 636 g mol^−1^ and 2.95 g cm^−3^, respectively, the corresponding volume of exGRc-Fe(III) is determined as *V*_exGRc-Fe(III)_ = 2.3 × 10^−14^ cm^3^[19,25]. If we divide this volume by the studied surface area (10^−8^ cm^2^), we obtain 23 nm. Since only the particles at the front side were counted, the final calculated thickness value *δ* should be equal to twice, i.e., 46 nm, which is quite consistent with the thickness values measured on some particles in Figure 4a.

In Figure 4b, Ag nanoparticles appear as polyhedrons with an apparent preferential location at the edge of exGRc-Fe(III) particles. A similar analysis as before was performed. The surface density of particles, *N*_Ag_ is 26 μm^−2^. From the size distribution in the insert and assuming a spherical shape of Ag nanoparticles, we obtained *V*_Ag_ = 4.2 × 10^−15^ cm^3^, a value approximately three times higher than for Au, consistent with the molar volume values, 10.3 and 10.2 cm^3^ mol^−1^ for Ag and Au, respectively. The corresponding *δ* value (44 nm) is very close to the one found above.

**Figure 5:**
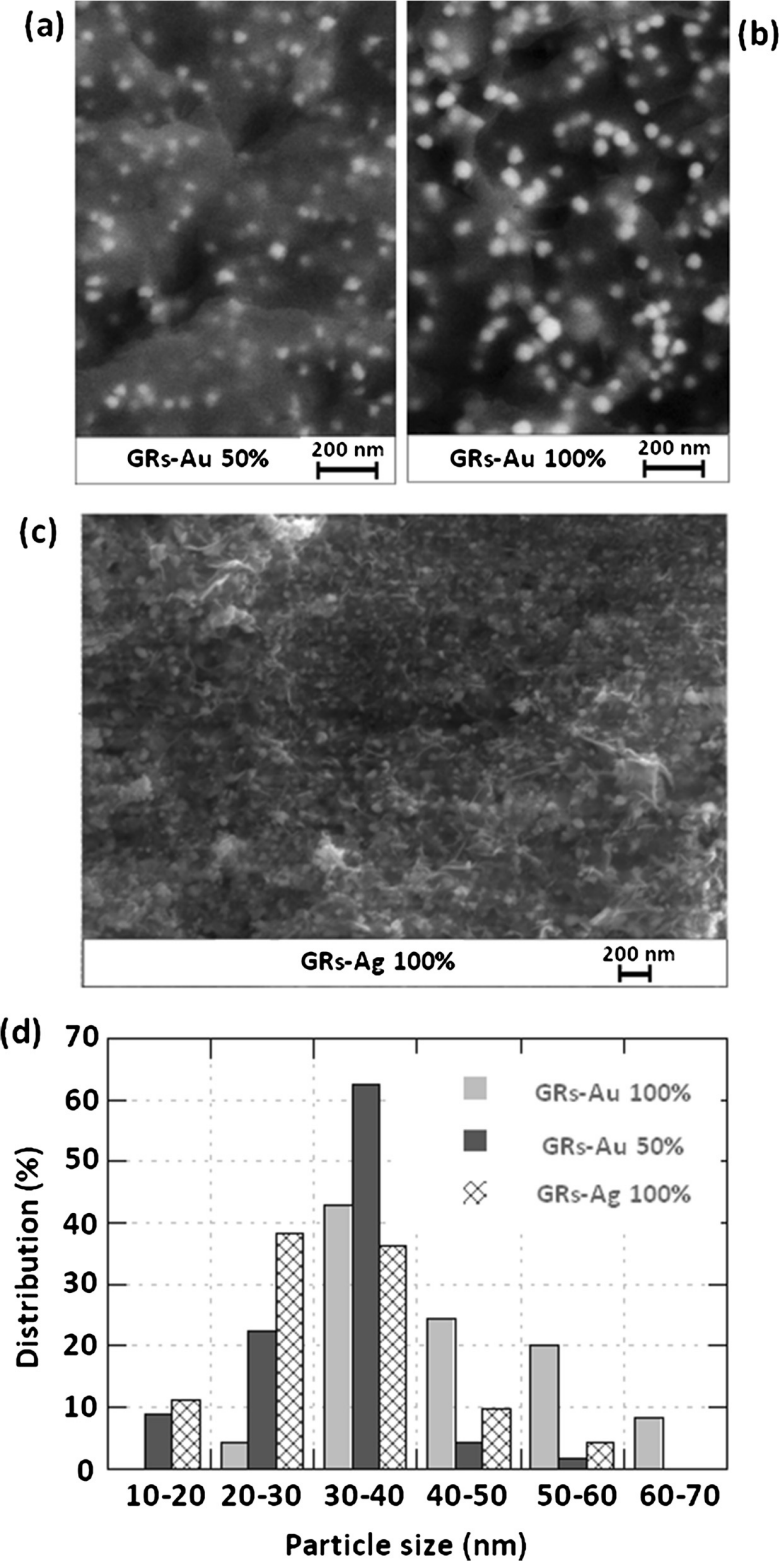
**Backscattered electron SEM microscopy pictures. **Solid samples obtained after interaction between (**a**) GRs and Au^III^, *R* = 50%, (**b**) GRs and Au^III^, *R* = 100% and (**c**) GRs and Ag^I^, *R* = 100%. (**d**) Size distribution histograms in (a) 3.5 μm^2^, 232 Au nanoparticles; (b) 3.5 μm^2^, 254 Au nanoparticles; and (c) 2 μm^2^, 135 Ag nanoparticles.

For experiments with sulfate green rust, in-lens mode analysis did not give satisfying results, since it was difficult to distinguish the metal nanoparticles and the thin exGRs-Fe(III) inorganic particles. Therefore, we report backscattered electron microscopy pictures (Figure 5). Au nanoparticles are clearly evidenced in Figure 5a,b, and we can also see the edges of some exGRs-Fe(III) particles. The surface density values obtained at *R* = 50% and at *R* = 100% are very close, at 67 and 73 μm^2^. The size distributions are given in Figure 5d; for *R* = 50%, the domain is quite narrow since 85% of the nanoparticles have sizes between 20 and 40 nm. The average size values are 32 and 43 nm; this result may suggest that the size of the particles decreases as lower and lower *R* values are chosen (from 100% to 0%). Since Ag has a lower molar mass than Au, the contrast displayed by Figure 5c is not well marked, but the Ag particles formed on exGRs-Fe(III) can still be analyzed. About 75% of the particles are in the 20 to 40 nm domain, the average size is 31 nm, and the surface density is 68 μm^−2^.

The whole previous results show that a green rust particle can be used as a micro-reactor for the synthesis of metal particles. The electrons consumed for the reduction of the soluble precursor to metal come from the oxidation of structural Fe^2+^ to structural Fe^3+^, which causes the progressive transformation of green rust to exGR-Fe(III) with no morphology change. The quantity of deposited metal is governed by the size of the GR particle. Actually, about one to ten metal nanoparticles on each inorganic particle are commonly observed.

**Figure 6:**
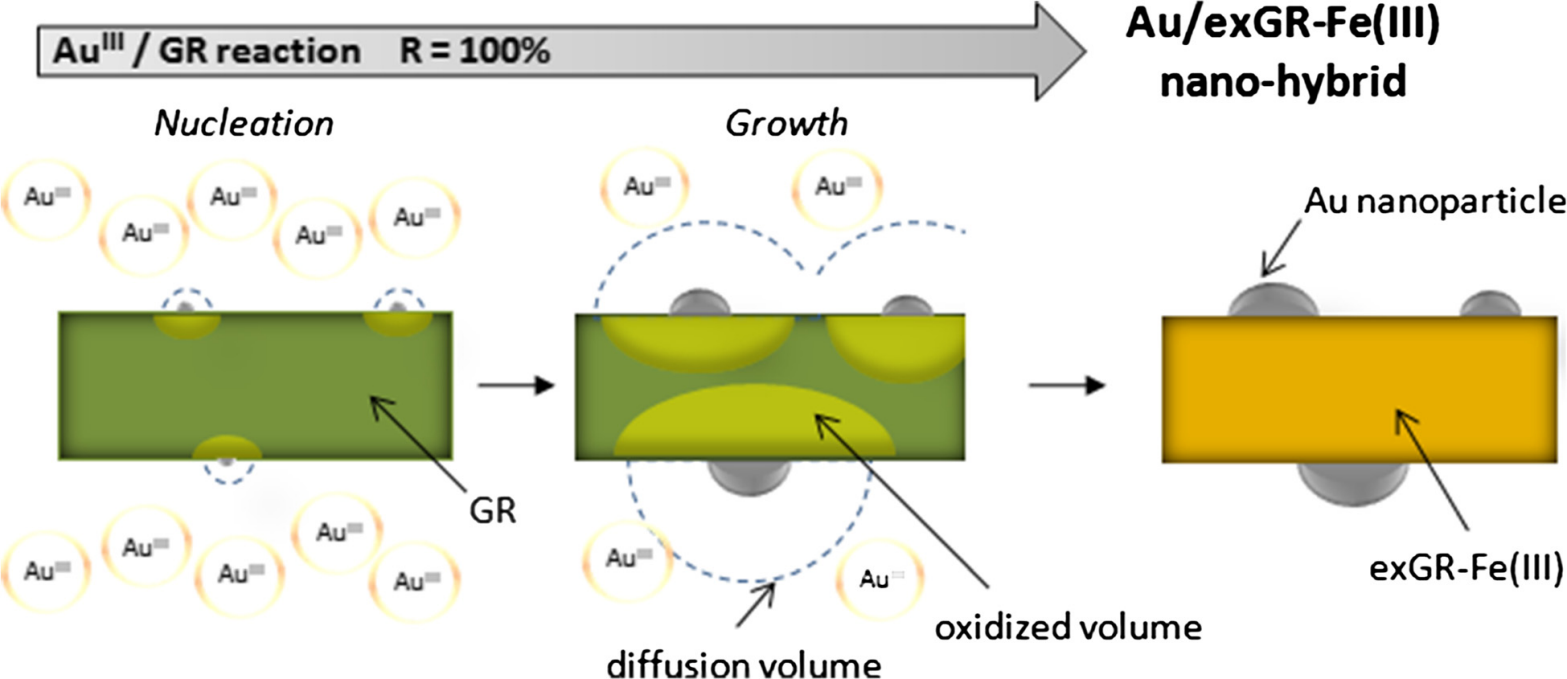
**Cross-sectional schematic of Au**^**III**^**/GR reaction.**

Figure 6 summarizes the reaction mechanisms occurring during the interaction between green rust and Au^III^ (it is similar in the case of Ag^I^). After the initial step of nucleation, the growth of gold clusters can be monitored by the diffusion of Au^III^ ions or by the transport of electrons from increasingly far Fe^II^ sites to the metal nanoparticle. Since there is no significant limitation during the formation of nanohybrids, the inorganic part must remain sufficiently conductive to allow the transport of the whole electrons from the bulk to the surface. The occurrence of exGR-Fe(III)* transient compounds (marked as ‘oxidized volume’ in Figure 6), keeping temporarily the conductive structure of green rust, may explain the observed high reaction rates; these exGR-Fe(III)* transient compounds were fully evidenced by voltammetry in our previous works [19,22]. Whatever the *R* values are, the samples display mass values that are in consistency with Equations 2 and 3. The metal loads that can be obtained from our method are between 0 and the maximal theoretical values, 25.2% for Au/exGRs-Fe(III), 29.2% for Au/exGRc-Fe(III), 35.6% for Ag/exGRs-Fe(III), and up to 40.4% for Ag/exGRc-Fe(III). These load values are very high and should even be increased after calcination to hematite *α*-Fe_2_O_3_.

With only one final separation step and the use of non-hazardous reagents, the synthesis of our metal/exGR-Fe(III) nanohybrids is very attractive. Due to their flat shape, the nanohybrids can be easily separated from a solution by filtration, either after their synthesis or after their operation as colloidal reagents. Moreover, their manipulation is very easy and relatively safe since mineral types such as iron compounds are generally fully biocompatible and metal nanoparticles are well attached to the inorganic matrices. The surface of inorganic and/or metal parts can be functionalized to target specific properties. The nanohybrids can be compacted to build permeable reactive membranes for remediation or disinfection treatments and heterogeneous catalysis. The formation of thin films by cast deposition, for example, may also be considered for the fabrication of modified (bio-) electrodes dedicated to analytical applications. If necessary, the inorganic part could even be partially or entirely removed by acidic or reducing treatments. This facile removal is attractive when the device requires metal nanoparticles only.

## Conclusion

The paper reports a new, simple, and fast (40 min) one-pot synthesis of supported Au and Ag nanoparticles in which a reactive Fe(II)-bearing green rust inorganic particle is used as an individual micro-reactor acting as both the reducing agent and the support for the resulting metal nanoparticles. The reaction of carbonate or sulfate green rusts with AuCl_4_^−^ or Ag(NH_3_)_2_^+^ involves the solid-state oxidation of green rust, and the reduction/precipitation onto the inorganic surface of Au or Ag metal. The resulting nanohybrids display a platy shape inorganic part, similar to the green rust precursor, supporting about one to ten metal nanoparticles which appear as flattened hemispheres (Au) or as polyhedrons (Ag). The size ranges are 10 to 60 nm for sulfate green rust and 20 to 120 nm for carbonate green rust. The field of potential applications of these nanohybrids is very large and deserves a careful exploration in the future.

## Competing interests

The authors declare that they have no competing interests.

## Authors’ contributions

SA and CP carried out the experiments. SA, CP and LL analyzed the data. LL developed the conceptual framework and supervised the whole work. LL and SA drafted the paper. All authors approved the final manuscript.
